# Perceived access to general and mental healthcare in primary care in Colombia during COVID-19: A cross-sectional study

**DOI:** 10.3389/fpubh.2022.896318

**Published:** 2022-09-07

**Authors:** Carlos Gómez-Restrepo, Magda Cepeda, William C. Torrey, Fernando Suarez-Obando, José Miguel Uribe-Restrepo, Sena Park, María Paula Jassir Acosta, Pablo Martínez Camblor, Sergio M. Castro, Jeny Aguilera-Cruz, Lilian González, Natalia Chaparro, Ana María Gómez-Gamez, Kathleen Bell, Lisa A. Marsch

**Affiliations:** ^1^Departamento de Epidemiología Clínica y Bioestadística, Pontificia Universidad Javeriana, Bogotá, Colombia; ^2^Hospital Universitario San Ignacio, Bogotá, Colombia; ^3^Department of Psychiatry, Geisel School of Medicine, Dartmouth College, Hanover, NH, United States; ^4^Insituto de Genética, Pontificia Universidad Javeriana, Bogotá, Colombia; ^5^Departamento de Psiquiatría y Salud Pública, Pontificia Universidad Javeriana, Bogotá, Colombia; ^6^Center for Technology and Behavioral Health, Dartmouth College, Lebanon, NH, United States

**Keywords:** mental health, primary health care, COVID-19, healthcare access, depression, unhealthy alcohol use, mental healthcare

## Abstract

**Introduction:**

The COVID-19 pandemic has had an impact both in general and mental healthcare, challenged the health systems worldwide, and affected their capacity to deliver essential health services. We aimed to describe perceived changes in ease of access to general and mental healthcare among patients with a diagnosis of depression and/or unhealthy alcohol use in Colombia.

**Methods:**

This study is embedded in the DIADA project, a multicenter implementation research study aimed at evaluating the integration of mental healthcare in primary care in Colombia. Between November 2020 and August 2021, we conducted a COVID-19 pandemic impact assessment in a cohort of participants with newly diagnosed depression and/or unhealthy alcohol use part of DIADA project. We assessed the ease of access and factors related to perceived ease of access to general or mental healthcare, during the COVID-19 pandemic.

**Results:**

836 participants completed the COVID-19 pandemic impact assessment. About 30% of participants considered their mental health to be worse during the pandemic and 84.3% perceived access to general healthcare to be worse during the pandemic. Most of participants (85.8%) were unable to assess access to mental health services, but a significant proportion considered it to be worse. Experiencing worse ease of access to general healthcare was more frequent among women, patients with diagnosis of depression, and patients with comorbidities. Experiencing worse ease of access to mental healthcare was more frequent among patients aged between 30 and 49.9 years, from socioeconomic status between 4 and 6, affiliated to the contributive social security regime, attending urban study sites, and those who perceived their mental health was worse during the pandemic.

**Discussion:**

Despite the overall perception of worse mental health during the pandemic, the use of mental healthcare was low compared to general healthcare. Ease of access was perceived to be worse compared to pre-pandemic. Ease of access and access were affected by geographical study site, socioeconomic status, age and gender. Our findings highlight the need for improved communication between patients and institutions, tailored strategies to adapt the healthcare provision to patients' characteristics, and continued efforts to strengthen the role of mental healthcare provision in primary care.

## Introduction

The COVID-19 pandemic challenged the provision of healthcare worldwide. Healthcare institutions re-organized and adapted to continue providing both COVID and non-COVID related care. They did so within government-imposed constraints to contain the pandemic spread, which included social distancing, lockdowns, and mobility restrictions. Some strategies used by healthcare institutions included implementing remote healthcare and the prioritization of services deemed as essential (1–4). The implementation of these strategies required adaptation and an accelerated learning curve for all the actors within the systems, but especially for health providers and patients, who had to take part in navigating new processes for healthcare provision and access.

The adjustments that institutions went through to continue providing healthcare within the constraints of the pandemic tested their preparedness for the use of technology in healthcare provision and the fluidity of their communication with their patients. It was not easy and several reports show that health institutions struggled to meet the healthcare and information needs of the patients (2, 5–8). For example, while the institutions required people to self-isolate and to practice social distancing, they provided limited and fragmented information about where and how patients could continue receiving non-COVID healthcare. Along with the fear of contagion, these factors compounded the burden among patients with making the decision whether their symptoms or health conditions were worthy of seeking any healthcare or postponing until unavoidable (5, 7–9). Mental healthcare is one service severely affected by this situation. Indeed, mental healthcare was often deemed as non-essential, resulting in numerous understaffed mental health units and care prioritized only for emergencies and critical cases (6, 8). This magnified already existing barriers in access to mental healthcare, where not only already diagnosed patients struggled to maintain their ongoing care but non-diagnosed patients were undetected, undiagnosed, and untreated. Along with the unprecedented societal, familial and economic burden of the pandemic, the lack of mental healthcare may have contributed to the large toll that mental health difficulties took on the population public health (10, 11).

The DIADA project is a multicenter implementation research project aimed at assessing the integration of a technology-based mental healthcare in six primary care sites in Colombia (12, 13). The model leverages technology and collaborative learning to improve detection, diagnosis and treatment of depression and unhealthy alcohol use. The model implementation was suspended at the time of the arrival of the pandemic in Colombia, in March 2020. At the time, the model had been in preparation and implementation for between 2 years and 6 months across the study sites. In this paper, we assess the perceived ease of access to general and mental healthcare during the pandemic among patients diagnosed with depression and/or unhealthy alcohol use during the model implementation at the study sites.

## Materials and methods

### Methods and design

This study is embedded in the DIADA project. Briefly, the DIADA project implementation was based on a modified stepped-wedge design, where the model was implemented at a new site approximately every 6 months starting on February 2018 through February 2020. The model design has been described elsewhere (12, 13). The project leverages technology and collaborative learning to integrate the model in healthcare provision by general practitioners through universal screening, diagnostic support, and healthcare providers' training in identifying and treating depression and/or unhealthy alcohol use. During the model implementation, we invited patients with newly diagnosed depression and unhealthy alcohol use to participate in a cohort for symptom follow-up during the year after diagnosis, with visits at the third, sixth, ninth, and 12 months. The in-person and by-phone follow-ups were conducted by trained research assistants. We suspended the model implementation on March 16th, 2020, due to the country-wide restrictions imposed to mitigate the COVID-19 pandemic spread. We continued the cohort follow-up remotely by phone. On November 2020, a COVID-19 impact survey was included in the scheduled follow-up questionnaire, provided that the patient agreed to answer it. Given the timing of the survey inclusion, it was applied at the 9 and 12 month follow-up call among the majority of the participants who were being followed. For patients that had already completed their year of follow-ups at the time of the survey inclusion, we requested IRB authorization and the patients' consent to contact them to complete the survey.

### Setting

The technology-based mental healthcare model was first implemented in February 2018 in a primary care center in Bogotá DC. Afterwards, it was implemented in rural Santa Rosa de Viterbo (August 2018), semi-rural Duitama (February 2019), rural Guasca (August 2019), and rural Soacha and Armero-Guayabal (February 2020).

### Participants

Participating patients were newly diagnosed adults (aged 18 years or older) with depression and/or unhealthy alcohol use, detected during consultation with a general practitioner in primary care. We excluded patients with a diagnosis of severe concomitant mental illness such as schizophrenia, bipolar disorder, depression with psychotic characteristics or who expressed suicidal intent. Patients intoxicated or with alcohol withdrawal symptoms who required a higher level of care (emergencies or hospital treatment) or who were unable to provide their informed consent, were not part of the study.

### Variables and measurements tools

The COVID-19 impact survey was developed by researchers of the NIMH U19 Scale-up Hubs (14). The instrument measures the local response to COVID-19 (1 item), exposure to COVID-19 (5 items), impact of COVID-19 (19 items) and access to mental and general health services (10 items). The impact of COVID-19 includes issues such as stigmatization, food insecurity, economic impact, mental health, and alcohol and drug use during the pandemic. We made minor modifications to the survey to add site-specific language and follow-up items to clarify responses. The questionnaire was implemented using REDCap electronic data capture hosted at Pontificia Universidad Javeriana (15, 16) and the research assistants registered the patients' responses through a tablet or computer interface.

### Bias

We attempted to minimize selection bias by building a standard follow-up procedure for contacting patients, including phone calls and standard SMS throughout the follow-up window.

### Outcome measurement

We assessed the perceived ease of access to general and mental healthcare with two questions, where responses options were: easier than before, same as before, more difficult than before, and non-applicable. The questions were prompted with “compared to before the quarantine in March 2020, getting mental (or general) healthcare within the COVID-19 context has been:”. We introduced the questions asking the patients to reflect on their experiences in obtaining general or mental healthcare, including access in any healthcare-related context, such as in-person appointments, emergency visits, phone calls and online services with a psychologist, psychiatrist, and/or a primary healthcare provider. The patients assessed the ease of access as non-applicable when they reported not having used or sought to use either service during the pandemic. For analysis purposes, we re-categorized the response alternatives as “same as” or “better than before”, “worse than before”, and “non-applicable”. The questions used in this module are shown in the Appendix 1.

### Sociodemographic and clinical factors

The study asked participants to report on sociodemographic and clinical factors during the recruitment and/or follow-up visits. Gender was registered as male or female. Age was calculated as the years between the date of birth and the date of answering the survey and categorized as 29.9 years or younger, between 30 and 49.9 years, between 50 and 69.9 years, and 70 years or older. Socioeconomic status (SES) (17) was re-categorized as rural SES 1–3, SES 4-6, no response. Social security affiliation was re-categorized as subsidized, contributive, no insurance, prepaid, complementary, or no response. Confirmed COVID-diagnosis was defined as a positive result of PCR as reported by the patient. Comorbidities were defined as having reported any diagnosed condition at recruitment (yes, no, no response). We asked the patients to assess their mental health during the pandemic compared to before, including whether it was worse, about the same, better than before, or no response. Baseline diagnosis corresponded to the diagnosis of depression or unhealthy alcohol use (alone or comorbid with depression) that brought the participant into the study. Symptom severity at baseline corresponded to the score obtained during the screening. For depression, we used the PHQ-9 questionnaire (18) and categorized scores as 0–9 (none to mild), 10–14 (moderate), 15–19 (moderate to severe), and 20–27 (severe). For unhealthy alcohol use, we used the AUDIT questionnaire (19) and categorized to 0–7 (none), 8–15 (mild), and 16–35 (moderate to severe).

### Statistical analysis

The dataset was downloaded and analyzed using the statistical software Stata 14.0 (20). Through data recruitment, a predefined process was implemented to assess and resolve missing information and variable outliers, by either recontacting the participant or verifying information with the corresponding research assistant. We conducted a descriptive analysis of sociodemographic and clinical factors and of the outcome variables (perception of ease of access). Qualitative variables were described as absolute and relative frequencies. Quantitative variables were described as medians and percentiles 25 and 75th. We compared the distribution of sociodemographic and clinical factors of the patients according to their perceived ease of access to either general or mental healthcare. We tested the statistical significance using the Fisher exact test and considered *p* values below 0.05 to be significant.

Our study was approved by the ethics committees of the Pontificia Universidad Javeriana in Colombia and Dartmouth College in the US, as well as by a Data and Safety Monitoring Board appointed by NIMH. All participants provided their written informed consent to participate in the study and gave their verbal informed consent prior to completing the COVID impact questionnaire.

This paper was written following the strengthening the reporting of observational studies in epidemiology (STROBE) recommendations for cross-sectional studies (21).

## Results

Out of 1,258 cohort participants, 836 participants were reachable and agreed to participate in the COVID-19 impact survey. Of these, 760 had a diagnosis of depression, and 76 had a diagnosis of unhealthy alcohol use with or without co-diagnosis of depression. Participants with a depression diagnosis were more likely to participate in the survey than participants with unhealthy alcohol use (68 vs. 56%). Table 1 shows the demographic characteristics of the population. Overall, 77% of the survey respondents were female, about half were aged between 50 and 69.9 years, and about 53% identified their ethnicity as “mestizo”. About half of the population belonged to socioeconomic status between 1 and 3 and were married or co-habitating. More than two thirds of the population had any comorbidity (77.8%). There were significant differences in sex, age and marital status distribution of the participants according to baseline diagnoses. Patients with depression diagnosis were mostly women (82.4%), aged between 50 and 69.9 years (50.7%) and married or cohabitating (46.7%), whereas patients with unhealthy alcohol use were primarily men (75%), aged between 18 and 29.9 years (51.3%), and single (47.4%).

**Table 1:** Characteristics of the study population, according to baseline diagnosis.

**Factors**	**Depression (*n* = 760/1,121)**	**Unhealthy alcohol use^b^ (*n* = 76/137)**	**Total (*n* = 836/1,258)**	***p*-value**
**Sex**				
Men	134 (17.6%)	57 (75%)	191 (22.8%)	<0.001
Women	626 (82.4%)	19 (25%)	645 (77.2%)	
**Age (years)**				
18-29.9	99 (13%)	39 (51.3%)	138 (16.5%)	<0.001
30-49.9	190 (25%)	18 (23.7%)	208 (24.9%)	
50-69.9	385 (50.7%)	15 (19.7%)	400 (47.8%)	
70-89.9	86 (11.3%)	4 (5.3%)	90 (10.8%)	
**Socioeconomic status**				
Rural	110 (14.5%)	10 (13.2%)	120 (14.4%)	0.542
SES 1–3	403 (53%)	36 (47.4%)	439 (52.5%)	
SES 4–6	239 (31.4%)	30 (39.5%)	269 (32.2%)	
NA/NR	8 (1.1%)	0 (0%)	8 (1%)	
**Social security affiliation**				
Subsidized	537 (70.7%)	49 (64.5%)	586 (70.1%)	0.479
Contributive	219 (28.8%)	27 (35.5%)	246 (29.4%)	
Other^a^	3 (0.4%)	0 (0%)	3 (0.4%)	
NA/NR	1 (0.1%)	0 (0%)	1 (0.1%)	
**Severity of depression symptoms, according to PHQ-9**				
None to mild	287 (37.8%)	46 (60.5%)	333 (39.8%)	<0.001
Moderate	290 (38.2%)	19 (25%)	309 (37%)	
Moderate to severe	139 (18.3%)	6 (7.9%)	145 (17.3%)	
Severe	44 (5.8%)	5 (6.6%)	49 (5.9%)	
**Severity of unhealthy alcohol use symptoms, according to AUDIT**				
None	737 (97%)	1 (1.3%)	738 (88.3%)	<0.001
Mild	14 (1.8%)	45 (59.2%)	59 (7.1%)	
Moderate to severe	9 (1.2%)	30 (39.5%)	39 (4.7%)	
**Setting of study site**				
Rural	167 (22%)	21 (27.6%)	188 (22.5%)	0.151
Urban	247 (32.5%)	29 (38.2%)	276 (33%)	
Semi-rural	346 (45.5%)	26 (34.2%)	372 (44.5%)	
**Confirmed COVID-diagnosis**				
No	680 (89.5%)	67 (88.2%)	747 (89.4%)	0.697
Yes	80 (10.5%)	9 (11.8%)	89 (10.6%)	
**Comorbidities**				
Yes	604 (79.5%)	46 (60.5%)	650 (77.8%)	<0.001
No	150 (19.7%)	30 (39.5%)	180 (21.5%)	
NA/NR	6 (0.8%)	0 (0%)	6 (0.7%)	
**Mental health during pandemic**				
Worse than before	236 (31.1%)	20 (26.3%)	256 (30.6%)	0.159
About the same	459 (60.4%)	51 (67.1%)	510 (61%)	
Better than before	64 (8.4%)	4 (5.3%)	68 (8.1%)	
NA/NR	1 (0.1%)	1 (1.3%)	2 (0.2%)	

NA/NR Not applicable/No response. SES Socioeconomic status.

^a^No insurance/Prepaid/Complementary.

^b^With or without depression.

In Table 2, we show the differences in the perception of ease of access to general and mental healthcare, according to sociodemographic and clinical factors. Regarding general healthcare, 84.3% of the patients assessed the ease of access to be worse and 7.36% considered it was the same or better than before the pandemic. Women were more likely to assess it as worse (86.7 vs. 76.3%, *p* < 0.001), as well as patients with a baseline diagnosis of depression compared to unhealthy alcohol use (85.7% vs. 70.7%, *p* < 0.001), and patients with comorbidities compared to patients without comorbidities (85.7% vs. 80%, *p* = 0.008).

**Table 2:** Distribution of sociodemographic and clinical factors related to perceived ease of access to general or mental healthcare.

**Factors**	**General healthcare**				**Mental healthcare**			
	**Same or better *n* = 61 (7.3%)**	**Worse *n* = 704 (84.3%)**	**Not applicable *n* = 70 (8.4%)**	***p*-value**	**Same or better *n* = 12 (1.4%)**	**Worse *n* = 107 (12.8%)**	**Not applicable *n* = 717 (85.8%)**	***P-*value**
**Sex**								
Men	14 (7.4%)	145 (76.3%)	31 (16.3%)	<0.001	3 (1.6%)	22 (11.5%)	166 (86.9%)	0.806
Women	47 (7.3%)	559 (86.7%)	39 (6%)		9 (1.4%)	85 (13.2%)	551 (85.4%)	
**Age (years)**								
18–29.9	11 (8%)	102 (73.9%)	25 (18.1%)	0.003	4 (2.9%)	20 (14.5%)	114 (82.6%)	0.059
30–49.9	15 (7.2%)	178 (86%)	14 (6.8%)		4 (1.9%)	35 (16.8%)	169 (81.3%)	
50–69.9	31 (7.8%)	346 (86.5%)	23 (5.8%)		3 (0.8%)	46 (11.5%)	351 (87.8%)	
70–89.9	4 (4.4%)	78 (86.7%)	8 (8.9%)		1 (1.1%)	6 (6.7%)	83 (92.2%)	
**Socioeconomic status**								
Rural	9 (7.5%)	97 (80.8%)	14 (11.7%)	0.018	0 (0%)	7 (5.8%)	113 (94.2%)	<0.001
SES 1-3	22 (5%)	375 (85.4%)	42 (9.6%)		3 (0.7%)	41 (9.3%)	395 (90%)	
SES 4-6	30 (11.2%)	224 (83.6%)	14 (5.2%)		9 (3.3%)	58 (21.6%)	202 (75.1%)	
NA/NR	0 (0%)	8 (100%)	0 (0%)		0 (0%)	1 (12.5%)	7 (87.5%)	
**Social security regime**								
Subsidized	30 (5.1%)	498 (85%)	58 (9.9%)	0.002	3 (0.5%)	49 (8.4%)	534 (91.1%)	<0.001
Contributive	31 (12.7%)	202 (82.4%)	12 (4.9%)		9 (3.7%)	58 (23.6%)	179 (72.8%)	
Other^a^	0 (0%)	3 (100%)	0 (0%)		0 (0%)	0 (0%)	3 (100%)	
NA/NR	0 (0%)	1 (100%)	0 (0%)		0 (0%)	0 (0%)	1 (100%)	
**Diagnosis**								
Depression	56 (7.4%)	651 (85.7%)	53 (7%)	<0.001	12 (1.6%)	99 (13%)	649 (85.4%)	0.667
Unhealthy alcohol use^b^	5 (6.7%)	53 (70.7%)	17 (22.7%)		0 (0%)	8 (10.5%)	68 (89.5%)	
**Setting of study site**								
Rural	8 (4.3%)	163 (86.7%)	17 (9%)	<0.001	1 (0.5%)	17 (9%)	170 (90.4%)	<0.001
Urban	35 (12.7%)	228 (82.9%)	12 (4.4%)		10 (3.6%)	67 (24.3%)	199 (72.1%)	
Semi-urban	18 (4.8%)	313 (84.1%)	41 (11%)		1 (0.3%)	23 (6.2%)	348 (93.5%)	
**Confirmed COVID-19 diagnosis**								
No	56 (7.5%)	622 (83.4%)	68 (9.1%)	0.05	11 (1.5%)	92 (12.3%)	644 (86.2%)	0.394
Yes	5 (5.6%)	82 (92.1%)	2 (2.2%)		1 (1.1%)	15 (16.9%)	73 (82%)	
**Comorbidities**								
Yes	50 (7.7%)	556 (85.7%)	43 (6.6%)	0.008	11 (1.7%)	85 (13.1%)	554 (85.2%)	0.741
No	11 (6.1%)	144 (80%)	25 (13.9%)		1 (0.6%)	22 (12.2%)	157 (87.2%)	
NA/NR	0 (0%)	4 (66.7%)	2 (33.3%)		0 (0%)	0 (0%)	6 (100%)	

NA/NR Not applicable/No response; SES Socioeconomic status.

^a^Other: No insurance/Prepaid/Complementary. ^b^With or without depression.

Regarding mental healthcare, 12.8% of the patients assessed the ease of access as worse and 1.4% assessed it as “same as or better than before”. The remaining 85.8% of the patients answered the question as “non-applicable”. Patients aged between 30 and 49.9 years were more likely to assess the ease of access to mental healthcare as worse (16.8%), along with patients from SES between 4 and 6 (21.6%), patients affiliated to the contributive social security regime (23.6%), attending urban study sites (24.3%), and patients who perceived their mental health was worse during the pandemic (27%). It is worth noting that, in most comparisons, patients were more likely to answer this question as “non-applicable” than “better as or same than before” or “worse than before” (Table 2).

**Figure 1:**
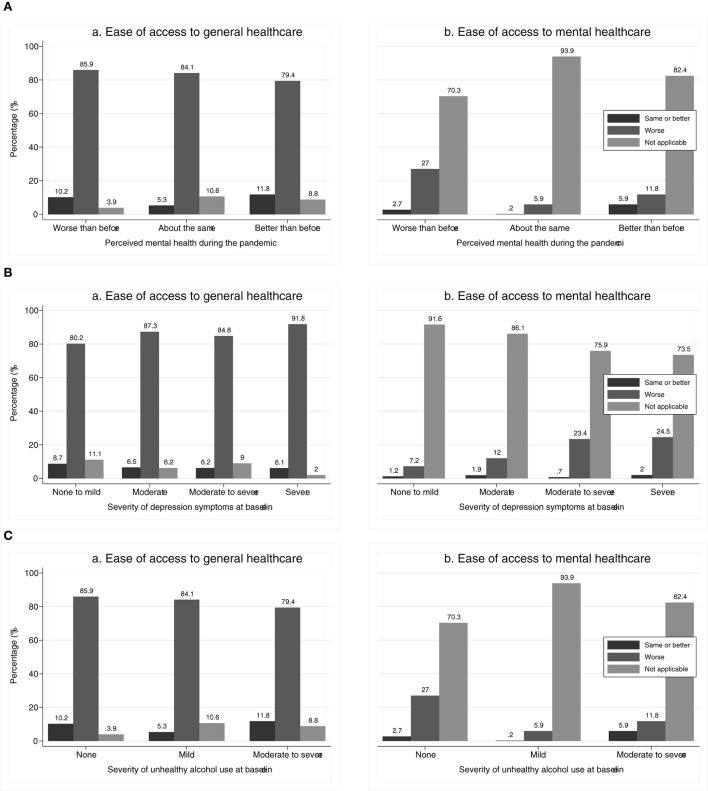
**(A)** Perceived ease of access (a) general and (b) mental healthcare during COVID-19, compared to before, according to perceived mental health during the pandemic^a^. **(B)** Perceived ease of access (a) general and (b) mental healthcare during COVID-19, compared to before, according to severity of depression symptoms at baseline^b^. **(C)** Perceived ease of access (a) general and (b) mental healthcare during COVID-19, compared to before, according to severity of unhealthy alcohol use at baseline^c^. ^a^Two participants who did not assess their perceived mental health during the pandemic assess ease of access to general healthcare as same as before and not applicable to mental healthcare. ^b^Severity of depression symptoms according to PHQ-9. ^c^Severity of unhealthy alcohol use according to AUDIT.

In Figure 1, we show the differences in the perception of ease of access to general and mental health according to (a) perceived mental health during the pandemic, (b) severity of depression symptoms at baseline, and (c) severity of unhealthy alcohol use at baseline. All differences were statistically significant (*p* < 0.001), except for the perceived ease of access to general healthcare according to severity of depression symptoms at baseline (*p* = 0.132).

## Discussion

Among 836 participants from a primary-care based cohort of patients with a diagnosis of depression and/or unhealthy alcohol use, the ease of access to general and mental healthcare was perceived as worse during the pandemic, compared to before the pandemic. Regarding access to mental healthcare, patients were more likely unable to access it and, among those who were able, they were more likely to perceive access as worse than before. For both general and mental healthcare, there were differences in the factors related to the perceived ease of access. For general healthcare, women, patients with baseline diagnosis of depression, and patients with any comorbidity were more likely to assess the ease of access as worse than before. In contrast, the ease of access for mental healthcare was more likely to be assessed as worse than before by patients aged between 30 and 49.9 years old, belonging to SES between 4 and 6, being affiliated to the contributive social security regime, and those who perceived that their mental health had worsened during the pandemic.

The challenges with access to general healthcare services, especially for non-COVID health conditions, have been described in multiple settings worldwide (3, 7, 22). Several factors have contributed to the quality and the quantity in healthcare access, including the diversion of resources toward the care of COVID patients, the prioritization of the health conditions deemed essential for healthcare provision, and the barriers to implementation of remote healthcare (such as by phone or online) (1, 3–5). Healthcare provision changes required health providers and patients to adapt to quickly changing steps in the process of healthcare and a fast-learning curve in healthcare systems' use of communication technology such as smartphones, computer programs, remote calls, email, and others (5). Therefore, in spite of the huge potential of technology use to improve the efficiency in healthcare processes such as education and information, triage, prescription refill, and consultation and therapy, the changes were a new barrier for patients with poor technology and internet literacy or poor access to technology, especially among patients from rural and semi-urban settings (23). Indeed, in a cross-sectional study with patients from primary care sites in Colombia, we showed that although nearly all the population had a cell phone, only 19.7% of them reported using the internet, 65% of them used the internet to look for health information, and only a third of participants used the phone to arrange a clinical appointment (24). Moreover, technology and internet literacy were lower in rural than in urban settings (24).

Health institutions struggled to adapt healthcare provision within the constraints of the pandemic, leaving patients needing care with multiple sources of uncertainty. For example, during the first days of the pandemic in Colombia, some study sites closed, appointments were canceled, and patients were referred to phone lines for information. However, some patients reported that when they called the lines were rarely answered. Although the emergencies rooms were available, patients dealt with the fear of COVID contagion in those sites. The intense public campaign for self-isolation and social distancing and the lack of clarity regarding the process changes implemented for healthcare provision left patients in the position of needing to decide whether they were candidates for healthcare (i.e., worthy of going to a hospital) and encumbering already crowded hospitals (1, 2, 6, 7, 9). This led to a number of patients refraining or postponing seeking any healthcare which, in some settings, has been correlated to avoidable mortality and poor outcomes for easily manageable health conditions (1, 6, 9). Therefore, the high prevalence of perceived worsening in ease of access to healthcare during the pandemic reflects the struggle of both institutions and patients to maintain fluid communication regarding the steps to both mitigate the pandemic spread and address ongoing healthcare needs.

We observed that women, patients with depression diagnosis, and those with any comorbidity were more likely to assess the ease of access to general healthcare as worse. First, this distribution reflects the actual demographic characteristics of the study sites population (12, 25), suggesting that they remained as the more likely to use the services during the pandemic, even if access was difficult. Nevertheless, the poor perception of ease of access suggests an unmet healthcare need. A large study conducted among European people aged older than 50 years-old reported that women were less likely than men to have their healthcare access postponed or denied (26). It has been reported that, during 2020, the worldwide prevalence of major depressive disorder was 3,152.9 per 100,000 population (95%CI 2, 722.5–3,654.5), which corresponds to an increase of 27.6% compared to before the COVID-19 pandemic (95%CI 25.1–30.3). Such increase was larger among women than among men [women: 29.8% (95%CI 27.3–32.5; men: 24.0% (95%CI 21.5–26.7))] (27). However, it has also been reported that women had large unmet healthcare needs during the pandemic, due to suppression of programs such as reproductive health and mental health, but also due to a larger risk of underemployment and caregiving roles (28, 29). Second, the fact that patients with any comorbidity were more likely to use available healthcare services, even if difficult, is explained by the presence of “chronic programs” or dedicated consultation for chronic health conditions at the study sites (such as hypertension and diabetes), where patients receive regular check-ups and prescription refill. This implied that patients with high baseline levels of healthcare utilization were seeking to get access. This finding was also observed among elderly European patients, where patients with poor overall health and high healthcare utilization had more unmet needs (26). In our context, at the beginning of the pandemic, some patients reported having bought their medication, as they were unable to get appointments in either chronic programs or regular consultation, although the situation eventually resolved. Finally, the finding that patients with more severe depression symptoms at baseline were more likely to use services, whereas patients with more severe symptoms of unhealthy alcohol use were less likely to use them, also reflects differences in the sex and age distribution between these diagnoses. Indeed, patients with depression were more likely to be middle to older aged (between 40 and 65 years-old) women, whereas patients with unhealthy alcohol use were more likely to be young-adult men. It also reflects the phenomenon that while severity of depression correlates to seeking help, patients with unhealthy alcohol use tend to seek less help.

A striking finding of our study is the low number of patients who were able to assess the ease of access for mental healthcare, in spite of being patients with diagnosis of either depression and/or unhealthy alcohol use. Less than 15% of patients assessed the ease of access to mental healthcare, and about 90% of those who did, assessed it as worse. These findings reflect the various barriers identified in access to mental healthcare in our settings (30), which became more evident in the context of the pandemic. First, the low availability of mental health trained healthcare providers, either specialized or not, worsened during the pandemic. For example, due to infection or because mental healthcare was often deemed non-essential, mental health units were understaffed and/or access was restricted to urgent or critical cases (6, 8, 31, 32). In our context, mostly psychologists and psychiatrists at secondary care services offer mental healthcare. However, whereas this option remained unchanged during the pandemic, patients with less severe symptoms at baseline were less likely to use the mental healthcare services, compared to those with more severe symptoms (Table 2). This suggests that either the patients, the institutions (including health insurers), or both, prioritized mental healthcare access for patients with greater symptom severity. Second, the lack of an established relationship between the patient and the healthcare institution led to less use of the services. The “Aging in the Time of COVID-19” study, a web-based survey conducted in 2020 among English speaking people from the US, showed that patients were more likely to access a healthcare provider and to receive medication during the pandemic if they had an established primary care provider relationship (29). These findings were similar to ours, where patients with more severe mental health symptoms were more likely to use mental healthcare services, probably for prescription refill. Third, the fragmentation, poor integration and unclear role of mental healthcare were reflected in the lack of specific strategies to maintain access during the pandemic. In our population, by the arrival of the pandemic in March 2020, we had been preparing and implementing the DIADA model of care between 2 years and 6 months at the study sites. Within a collaborative learning and technology-based model, we worked with the primary care study sites to integrate mental healthcare into their healthcare provision processes. However, most of the patients were unable to assess the access to mental healthcare, implying that either they do not yet consider general healthcare a source of mental healthcare or that they got a referral from their general practitioners, but were not able to navigate the system toward specialized mental healthcare or the mental healthcare received was not satisfactory. For example, some patients complained because the appointment was focused only on prescription refill. Indeed, among the patients who perceived their mental health worsened during the pandemic (255/836, 30%), only about 30% of them accessed mental healthcare but 96.1% reported having used general healthcare. In sum, these findings highlight the need for a continued effort to address the existing barriers to reduce the gaps in mental healthcare access: in the patients' expectations regarding the role of primary care in their mental healthcare, in the perceived role of primary care institutions and general practitioners for mental healthcare provision, and in the efforts by insurers and institutions to enhance the integration across healthcare levels for continued mental healthcare access.

Older adults (aged older than 50 years), patients belonging to SES between 1 and 3, affiliated with the subsidized social security regime, and from rural sites, were less likely to assess access to mental healthcare services. Similar findings have been reported in other settings. For example, in a study among pregnant participants in Massachusetts, those of color (Black, Asian, Multiracial, and/or Hispanic/Latino/a) were more likely to report experienced barriers in their mental healthcare during the pandemic (33). Structural barriers and healthcare access restrictions and policies in relation to immigrants affected their mental and physical health and their probability of seeking and/or actually receiving healthcare during the pandemic (34, 35). In contrast, patients with higher income tend to be more likely to seek and navigate services to gain access to a service. The “Aging in the Time of COVID-19” study showed that the access to medication was higher among older patients with a higher income, but lower among patients with caregiving responsibilities and social isolation (29). Besides structural barriers for access among underserved and poor population (7, 29, 34), lack of education and low technology and internet literacy in this population may also explain access differences. Low education is associated with lower recognition of mental health symptoms (30, 33) and lower technology and internet literacy (24), factors that negatively impact awareness and access to remote healthcare. Finally, large differences in technology and internet access and in the geographical distribution of healthcare professionals explain the differences found in the use and the perceived ease of access to mental healthcare between rural and urban patients. Living in urban settings was considered a potential barrier for healthcare access due to the stricter enforcement of isolation and lockdowns, but access challenges were mitigated by broadband access allowing remote healthcare. COVID-19 restrictions are less strictly enforced in rural areas, but the remote healthcare solutions are less useful (22, 23). In Colombian rural settings, technology and internet access is still difficult with insufficient broadband and low use of smartphones (24). In addition, the rural sites in our study do not have local psychologists and psychiatrists, so the patients must travel to cities nearby for their regular appointments, which increases out of pocket costs and requires the investment of time. These obstacles already discouraged patients to seek mental healthcare with specialized professionals in pre-pandemic time and, during the pandemic, it may have worsened, due to the mobility restrictions, the economic uncertainty, and communication issues between institutions and patients (8).

In Colombia, the affiliation to the healthcare system is mandatory through three regimes: contributive, subsidized, and special regimes (e.g., Military, Professors, and Indigenous). The affiliation occurs through health promoting institutions (EPS, in Spanish), which are mainly private. Additionally, a complementary prepaid regime is accessible through a premium. Healthcare is provided through health provider institutions (IPS, in Spanish), which can also be private or public. IPS are categorized according to the healthcare complexity level they are authorized to provide. The primary care level is the entry point to the health system. Although there are not restrictions to provide non-specialized mental healthcare in primary care, except for specific programs for health promotion and disease prevention (e.g., for physical activity promotion), mental healthcare is provided only by psychologists and psychiatrists at secondary and tertiary level of care. This implies that either a general practitioner or other specialist must refer the patient for specialized mental healthcare. Both psychiatrists and psychologists can implement a treatment plan based on therapy. Virtually, any medical doctor can prescribe psychiatric medications. Nevertheless, in practice, only psychiatrists do so and, for chronic use, patients must regularly attend an appointment for prescription renewal.

The pandemic proved a time for testing the adaptation preparedness of the health systems and institutions and the strength of the relation between patients and healthcare institutions. General healthcare and mental healthcare were both affected by unclear and inequitable adaptations and communication strategies. Although technology is a useful tool for adaptation and continued care, the evidence suggests it is not a one-size-fits-all tool and it requires both communication with, and adaptation to, the population resources and needs. We reflected on what the COVID impact survey tells us about the DIADA model of technology-enhanced depression and unhealthy alcohol use care in primary care. The DIADA model improves patients' access to mental healthcare (12, 25), but with COVID-19 the institutions struggled to maintain the integration of mental healthcare, due to several factors. First, although our model includes a universal screening strategy for depression and unhealthy alcohol use in the waiting rooms in primary care, this step was not feasible with site closures. Consequently, the diagnosis relied on the ability of the providers to identify the patients with mental health conditions and on the patients explicitly seeking mental healthcare. Second, the fact that the first step of our model required that the patient was physically at the primary care site will continue to be a barrier to patient identification to the extent that remote healthcare remains the standard of care for a number of health conditions (22, 23). This implies that the model must adapt to make it sustainable and acceptable through remote care. Third, our model leveraged technology and a collaborative learning strategy to train and support general practitioners to provide mental healthcare in primary care. For this to be effective, Colombian healthcare must strengthen the perception by health insurers, institutions and general practitioners of their key role in mental healthcare provision. Within the Colombian healthcare system, primary care providers continued care for multiple conditions, through programs and plans, encompassing processes ranging from education and treatment to health promotion (e.g., prenatal care) to primary and secondary prevention (e.g., vaccination and chronic programs). For example, even though the follow-up calls we implemented for symptoms assessment were not aimed as therapeutic interventions, our participants often expressed these were a space for relief and wellbeing, as they felt heard and cared for. Not only patients with diagnosed mental health conditions but also the entire base of clients from primary care will benefit from leveraging this regulatory framework and the benefits of technology to promote health and prevent disease through improved mental health (1). Fourth, we trained general practitioners based on a collaborative learning approach to provide mental health interventions depending on the severity of patients' symptoms. Yet, patients who required specialized care often mentioned barriers for access, including lack of psychologists and psychiatrists, a complicated process to access prescribed medications, and transportation to nearby towns to attend appointments. In the Colombian healthcare system, these issues arose partially due to financial and logistical priorities determined by health insurers. Therefore, health insurers should be key stakeholders for the adoption and implementation of our model in order to meet increased demand of mental healthcare in primary care centers. Finally, although our model helped to identify and increase the number of patients requiring mental healthcare in primary care, patients who accessed it during COVID-19 were those with more severe symptoms. These findings were also observed in a systematic review that reported that healthcare utilization decreased by about one third during the pandemic, especially for people at the milder spectrum of an illness (36). Although the authors consider these findings partly reflecting a reduction in over-diagnoses and over-treatment, these findings may also indicate the amount of unmet needs in healthcare and, consequently, relate to the increase of preventable non-COVID morbidity and mortality (9, 36) and the large toll mental health difficulties have had on public health worldwide. Therefore, health systems and institutions could strengthen their efforts to help patients develop awareness about their mental health, design and implement innovative community-tailored strategies to maintain the healthcare provision (including education and information), and find efficient and fast communication ways to help patients navigate the healthcare process.

Our study has some limitations. First, given its cross-sectional nature, it is unclear whether the ease of access to care actually changed during the pandemic. If the patients did not often use the services prior to the pandemic, they may have an unclear idea of how it actually changed. Second, we applied the COVID impact questionnaire between November 2020 and August 2021, spanning the second and third waves of the pandemic in Colombia. Therefore, the collected information reflects the experience of the patients up to the time of the survey, which may have been different in between the evaluation period, given all the adaptations that the healthcare institutions went through. Third, the participation rate in the survey was higher among patients with depression than among patients with unhealthy alcohol use. Therefore, the perceived worsening in ease of access to general and mental healthcare access reflect mostly the experience of the patients with depression, who were mainly women and patients with comorbidities. Nevertheless, the differences in participation according to diagnosis also reflect the differences in the demographic characteristics between these groups, where patients with unhealthy alcohol use were mostly young adult men. Overall, we found these patients were challenging to reach in spite our efforts to locate them. Frequently, their phone numbers had been canceled, which was likely a consequence of economic uncertainty. Given their demographic characteristics, we expect their experience would likely have been that of patients without comorbidities and who, compared to patients with depression, were less likely to use both general and mental healthcare services.

Finally, we did not explore reasons underlying the perception of worse access. Thus, we can only speculate based on the experience of other settings regarding access barriers throughout the pandemic, the information informally provided by the participants during the survey, and the dialogue with the hospitals' leaders. Nevertheless, our findings align with findings of other studies, adding valuable evidence regarding how patients experience healthcare access during the pandemic. To our knowledge, this is the first study that assesses this phenomenon in Colombia, a middle-income country located in Latin America, a region that experiences a large inequity in general and mental healthcare access and a large public health burden both by the pandemic and by mental health deterioration.

In conclusion, Colombian primary care patients diagnosed with depression and/or unhealthy alcohol use experienced worsened general and mental healthcare ease of access during the pandemic, compared to before the pandemic. Patients were unlikely to use mental healthcare services, which correlated to being low SES, affiliated with a subsidized social security regime, and attending a rural study site. The low use and the predominant perception of worsening access to general and mental healthcare reflect issues in the ability of the healthcare systems to adapt the care provision to their clients' resources, abilities and needs and the lack of working and standardized communication strategies between institutions and patients. These findings are not unique to our population, as the pandemic took a large toll in public health worldwide not only due to COVID-19 cases, but also due to unattended needs in non-COVID-19 health conditions. Our findings provide valuable evidence about factors that can be addressed in order to reduce the barriers and inequity in general and mental healthcare access in primary care among population from Colombia and Latin America.

## Data availability statement

Raw anonymized data will be made available upon request. Requests to access the datasets should be directed to MC, macepeda@javeriana.edu.co.

## Ethics statement

The studies involving human participants were reviewed and approved by Ethics Committees of the Pontificia Universidad Javeriana in Colombia and Dartmouth College in the US, as well as by a Data and Safety Monitoring Board appointed by NIMH. The patients/participants provided their written informed consent to participate in this study.

## Author contributions

CG-R, MC, WT, FS-O, JU-R, SC, LM contributed to conception and design of the study. MC, SP, MJ, JA-C, LG, NC, AG-G, LM contributed to data collection and review. MC and JA-C organized the database. MC wrote the first draft of the manuscript. CG-R, WT, FS-O, JU-R, SP, MJ, PM, KB, LM wrote sections of the manuscript. All authors contributed to manuscript revision, read, and approved the submitted version.

## Funding

Research reported here was funded under award number 1U19MH109988 by the National Institute of Mental Health of the National Institutes of Health (NIH) (principal investigators: LM and CG-R).

## Conflict of interest

Author LM is affiliated with the business that developed the mobile intervention platform used in this research. This relationship is extensively managed by author LM and her academic institution. The remaining authors declare that the research was conducted in the absence of any commercial or financial relationships that could be construed as a potential conflict of interest.

## Publisher's note

All claims expressed in this article are solely those of the authors and do not necessarily represent those of their affiliated organizations, or those of the publisher, the editors and the reviewers. Any product that may be evaluated in this article, or claim that may be made by its manufacturer, is not guaranteed or endorsed by the publisher.

## Author disclaimer

The contents are solely the opinion of the authors and do not necessarily represent the views of the NIH or the U.S. government.
