# Supplementary material for: Perceived access to general and mental healthcare in primary care in Colombia during COVID-19: A cross-sectional study
Source: Front Public Health. 2022 Sep 7;10:896318. doi: 10.3389/fpubh.2022.896318 (PMC9490130; doi:10.3389/fpubh.2022.896318)
Supplement: Supplementary file 1 [file Data_Sheet_1.PDF]

Appendix 1. Questions of the module Perceived ease of Access to general and mental healthcare.

Español: Las siguientes preguntas son un extracto del cuestionario empleado para evaluar el impacto de COVID-19 en participantes del proyecto DIADA y desarrollado por NIMH U19 Scale-Up Hubs - COVID-19 Metric Working Group.

Impacto en la salud mental

Fuente: Informado por Youth Readiness Intervention Recruitment Documents
Citation: Betancourt TS, McBain R, Newnham EA, et al. A behavioral intervention for war-affected youth in Sierra Leone: a randomized controlled trial. J Am Acad Child Adolesc Psychiatry. 2014;53(12):1288-1297. doi:10.1016/j.jaac.2014.09.011.. Disponible en:

MH1	Comparado con antes del inicio de la cuarentena en marzo de 2020, ¿cómo describiría su salud mental? Diría que es:	Peor que antes	0	
		Más o menos lo mismo que antes	1	
		Mejor que antes	2	
		Incapaz de responder	99	

Acceso a los Servicios de Salud Mental: Impacto en el acceso a los servicios de salud mental

Fuente: Modificada del Social Context of the Ebola Epidemic Survey, Wave 3, Section 15. Ebola hardships and daily hassles
Citation: Betancourt TS, Brennan RT, Vinck P,VanderWeele TJ, Spencer-Walters D, Jeong J, et al. (2016) Associations between Mental Health and Ebola-Related Health Behaviors: A Regionally Representative Cross-sectional Survey in Post-conflict Sierra Leone. PLoS Med 13(8): e1002073.doi:10.1371/journal.pmed.1002073. Available at:
https://journals.plos.org/plosmedicine/article/file?id=10.1371/journal.pmed.1002073&type=printable

ACC0	**INTRO: Ahora nos gustaría preguntar sobre cómo ha cambiado su acceso a la atención médica en salud mental debido al coronavirus/COVID-19.**<br><br>**Desde el inicio de la cuarentena en marzo de 2020, ¿cómo ha cambiado su capacidad para obtener atención en salud mental? La atención en salud mental incluye visitas en persona, llamadas telefónicas y servicios en línea con un psicólogo, psiquiatra o proveedor de atención primaria. Esto también incluye visitas a la sala de emergencias.**		
ACC1	Comparado con antes del inicio de la cuarentena en marzo de 2020, obtener atención en salud mental en el contexto de coronavirus/COVID-19 ha sido:	Más fácil	1
		Igual	2
		Más difícil	3
		No aplicable	99
ACC2	Comparado con antes del inicio de la cuarentena en marzo de 2020, obtener atención médica en general en el contexto de coronavirus/COVID-19 ha sido:	Más fácil	1
		Igual	2
		Más difícil	3

		No aplicable	99

<u>English: The following questions are an excerpt of the questionnaire used to assess the COVID-19 impact among participants of the DIADA Project and developed by the NIMH U19 Scale-Up Hubs - COVID-19 Metric Working Group.</u>

Impact on Mental Health

Source: Informed by Youth Readiness Intervention Recruitment Documents
Citation: Betancourt TS, McBain R, Newnham EA, et al. A behavioral intervention for war-affected youth in Sierra Leone: a randomized controlled trial. J Am Acad Child Adolesc Psychiatry. 2014;53(12):1288-1297. doi:10.1016/j.jaac.2014.09.011. Available from:

MH1	Compared to before the lockdown in March 2020, how would you describe your psychological health now? Would you say it is:	Worse than before	0	
		About the same as before	1	
		Better than before	2	
		Unable to answer	99	

Access to Mental Health Services: Impact on Access to Mental Health Services

Source: Modified from the Social Context of the Ebola Epidemic Survey, Wave 3, Section 15. Ebola hardships and daily hassles
Citation: Betancourt TS, Brennan RT, Vinck P,VanderWeele TJ, Spencer-Walters D, Jeong J, et al. (2016) Associations between Mental Health and Ebola-Related Health Behaviors: A Regionally Representative Cross-sectional Survey in Post-conflict Sierra Leone. PLoS Med 13(8): e1002073.doi:10.1371/journal.pmed.1002073. Available at:
https://journals.plos.org/plosmedicine/article/file?id=10.1371/journal.pmed.1002073&type=printable

ACC0	**INTRO: Now we would like to ask about how your access to psychological healthcare changed due to the coronavirus/COVID-19.**<br><br>**Since the lockdown in March 2020, how has your ability to obtain psychological health care changed? Psychological health care includes in-person visits, phone calls and online services with a psychologist, psychiatrist, or a primary care provider. This also includes emergency room visits.**		
ACC1	Compared to before the lockdown in March 2020, obtaining psychological health care needed in the context of coronavirus/COVID-19 has been:	Easier	1
		The same	2
		More difficult	3
		Not applicable	99
ACC2	Compared to before the lockdown in March 2020, obtaining general health care needed in the context of coronavirus/COVID-19 has been:	Easier	1
		The same	2
		More difficult	3

		Not applicable	99
